# A Multichannel Conflict-Free Mac Protocol for Enhancing RPMA Scalability

**DOI:** 10.3390/s23239363

**Published:** 2023-11-23

**Authors:** Enas Ali Alsaeedi, Fatma Bouabdallah

**Affiliations:** Faculty of Computing and Information Technology, King Abdelaziz University, Jeddah 21589, Saudi Arabia; emohammedsaeedalsaeedi@stu.kau.edu.sa

**Keywords:** internet of things (IoT), Low Power Wide Area (LPWA), Random Phase Multiple Access (RPMA), protocol, scalability, multichannel

## Abstract

The internet of things (IoT) revolutionized human life, whereby a large number of interrelated devices are connected to exchange data in order to accomplish many tasks, leading to the rapid growth of connected devices, reaching the tens of billions. The Low Power Wide Area (LPWA) protocols paradigm has emerged to satisfy the IoT application requirements, especially in terms of long-range communication and low power consumption. However, LPWA technologies still do not completely meet the scalability requirement of IoT applications. The main critical issues are the restrictive duty cycle regulations of the sub-GHz band in which most LPWA technologies operate, as well as the random access to the medium. Ingenu Random Phase Multiple Access (RPMA) is an LPWA technology that uses the 2.4 GHz band that is not subject to the duty cycle constraint. Furthermore, RPMA uses Direct-Sequence Spread Spectrum (DSSS) as a modulation technique; hence, it is an excellent candidate technology for handling scalable LPWA networks. In this paper, we perform mathematical and simulation analysis to assess RPMA scalability and the factors that affect it, especially when all the available channels are used. The results indicate that RPMA has impressive scalability. Indeed, by taking advantage of the multichannel feature in RPMA, the network capacity can be increased by up to 38 times. Aditionally, randomly selecting the Spreading Factors (SF) degrades the network scalability, as working on higher SFs will increase the probability of collision. Thus, we proposed an SF distribution algorithm that ensures effective packet delivery with minimum collision.

## 1. Introduction

Increasingly, companies in different sectors of industry are using IoT to operate more efficiently, provide improved customer service, enhance decision-making in order to raise the value of their business. Thus, many solutions based on IoT are suggested in a variety of industries, namely education, logistics, health, or even everyday use [1]. Indeed, IoT represents a revolution in the communications domain [2]. Hence, the number of IoT devices worldwide is expected to reach more than 25 billion connected sensors by 2030 [3].

However, IoT devices need more support regarding communication requirements, particularly coverage, energy budget, and deployment costs, which may limit the IoT applications’ performance and throughput [4]. Thus, it is essential to find proper technologies that meet IoT requirements. In the beginning, IoT relied on traditional technologies such as Bluetooth, Zigbee, Wi-Fi, and so on, which succeeded in providing high-speed and reliable transmissions. On the other hand, they demand high deployment costs and energy consumption, as in Wi-Fi and Bluetooth, or they provide short-range coverage of connected devices with lower energy consumption, as in Zigbee and Bluetooth Low Energy (BLE) [5]. Hence, they do not offer efficient solutions to long-range communication that require the devices to operate at low power in order to live for years [2,4].

In this context, the Low Power Wide Area (LPWA) protocols paradigm has emerged to fill the gaps of traditional technologies by handling the requirements of IoT applications [1]. LPWA is mainly designed to provide high coverage, low energy consumption, and low deployment costs. However, LPWA usually satisfies these requirements at the expense of latency and throughput. Many technologies have emerged under the LPWA family, such as LoRa, Ingenu, Sigfox, Dash7, Weightless, Telensa, etc. Each LPWA technology has its own properties based on the deployment scenario and the selected supplier specification. That being said, all LPWA technologies share four main features: comprehensive coverage, low cost, lower power consumption, and scalability. However, a tradeoff exists between having a scalable network and keeping it simple and cheap [4].

Many works in the literature address the scalability in LPWA technologies, particularly in LoRa and Sigfox [6,7,8,9]. These works succeed in handling the scalability in LPWA but within a limited range. Hence, they do not satisfy the requirement of the explosive growth of IoT applications, especially regarding the number of connected devices. Indeed, one of the main scalability constraints is the restrictive regulation of the duty cycle on the sub-GHz Industrial, Scientific, and Medical (ISM) band. Hence, bypassing such constraints will inevitably help to build large IoT networks with better performance.

Ingenu Random Phase Multiple Access (RPMA) is an LPWA technology that works on the 2.4 GHz band without the duty cycle restriction. Consequently, RPMA has high capacity and high coverage. Thus, it can leveraged to conceive a highly scalable protocol for IoT communication. In particular, RPMA uses the 2.4 GHz band with a bandwidth of 80 MHz. Furthermore, the Direct-Sequence Spread Spectrum (DSSS) signal of RPMA is of width 1 MHz. Hence, 40 distinct channels of 1 MHz can be defined. Mainly, RPMA exploits 1 MHz as a guard band to separate between channels. Thus, deploying 1 MHz as channel bandwidth in addition to 1 MHz as guard band will result in 40 used channels in an 80 MHz band. Then, RPMA partitions these channels in frequencies where the Wi-Fi channels are not typically deployed, hence avoiding interfering with Wi-Fi, which works in the same 2.4 GHz band [10]. In this paper, we perform a mathematical analysis to evaluate RPMA scalability based on collision probability. Moreover, we simulate RPMA using MATLAB. The results indicate that operating on higher spreading factors will increase the collision probability. In general, any increase in collision probability will reflect a decrease in Packet Delivery Ratio (PDR) and network throughput and vice versa. Thus, collision probability has a primary role in RPMA scalability. Furthermore, we prove that RPMA’s ability to demodulate overlapping signals helps to highly reduce collision probability. In addition to that, employing the multichannel feature in RPMA will enhance RPMA scalability up to 38 times.

In order to reduce the effect of higher SFs on RPMA scalability while ensuring a satisfactory packet delivery, we proposed an SFs distribution algorithm. Our main goal is to reduce using the higher SFs without compromising the packet delivery. In other words, high SFs will be assigned only to far nodes in order to avoid losing packets. We studied three scenarios. The first one is fully random, where nodes randomly select the SF without considering the SF coverage. The second scenario, as opposed to the first one, is deterministic; where nodes within a particular range must choose a specific SF. Specifically, the lowest eligible SF that can cover the node range. The third scenario is partially random, where nodes can choose any eligible SFs that can cover the node range. The best results were obtained by the second scenario, as it has the lowest packet error rate. Indeed, in the first scenario, SFs coverage is not considered, leading to the loss of many packets, as the randomness in selecting the SF increases the use of higher SFs and hence increases the collisions. Although the third scenario considers the SFs coverage, the randomness of choosing among eligible SFs still increases the collision probability compared to the second scenario.

The rest of the paper is organized as follows. Section 2 provides a general idea about LPWA specifications. Section 3 reviews related work discussing the scalability of LPWA technologies in general and the one of RPMA in specific. Section 4 introduces an overview of the RPMA protocol. Section 5 provides a mathematical analysis of RPMA probability of collision. Section 6 presents and discusses the simulation results. Section 7 discusses the SF distribution algorithm. Finally, the paper concludes in Section 8.

## 2. LPWA Technologies

The Low Power Wide Area (LPWA) protocols paradigm has emerged to satisfy the IoT application requirements. LPWA technologies are communication protocols that provide long-range communication to transmit low data rates while consuming low power [4]. Indeed, although LPWA technologies succeed to achieve long-range and low energy consumption, they accomplish a low data rate (tens of kilobits per second) and high latency (seconds or minutes) [1]. Accordingly, LPWA technologies are proper for massive IoT applications, demanding low power consumption, low cost, low data rate, and delay-tolerant [1], e.g., smart cities, agriculture, transport, logistics, etc. [2]. Several LPWA solutions have been introduced, such as LoRa, Sigfox, Ingenu, etc. LPWA technologies may have different deployment scenarios according to the policy and standards that the supplier followed. However, all LPWA technologies shared four target features, namely: long-range, low-power, low-cost, and scalability. In this section, we want to highlight the main features of LPWA and then explore some of LPWA’s technologies. Each LPWA technology tries to apply different mechanisms to satisfy the main LPWA specifications.

For instance, to cover a wide area with excellent signal propagation where the deployment environment, band, and modulation technique affect the communication range, most LPWA technologies use the sub-GHz band to provide reliable communication with a low power budget. In addition, using the sub-GHz band can help to decrease the possibility of interference between various communication techniques. Indeed, using the sub-GHz band is less likely to be congestive compared to the 2.4 GHz band used in other technologies such as Bluetooth, Zigbee, and Wi-Fi. However, Ingenu RPMA technology adopted the 2.4 GHz band, which has more relaxed spectrum regulations regarding the duty cycle and transmission power. Moreover, LPWA technologies adopted two modulation techniques in order to achieve long-range communication, namely: narrowband (assign very narrowband for each carrier) and spread spectrum techniques (a narrowband signal spreads over a broader frequency band) [1,4].

On the other hand, LPWA technologies usually run on a cheap power source and cannot be easily replaced. So, there is a need for ten years or more of battery life. Accordingly, most LPWA technologies adopt star topology where the sensor nodes are directly connected to the base station. In this way, they provide quick access and significantly save communication energy as they avoid consuming the energy of forwarder nodes in multi-hop communication. In addition, LPWA technologies applied a duty cycling mechanism to further save energy. Additionally, most LPWA technologies use ALOHA, a simple random access MAC protocol that does not require carrier listening, which may consume extra energy. Moreover, they keep a simple design of end devices by offloading complicated jobs to the base stations. Hence, the transceiver will be simple and low-cost [1,4].

To succeed, LPWA technologies must address wide-range and compete with other technologies in the domain, such as cellular networks and short-range wireless LAN networks. Consequently, they aim at connecting many end devices with a cost of hardware below USD 5.00. Using star topology, simple MAC protocols, and offloading techniques helps companies manufacture low-cost devices and deploy them [1]. Furthermore, besides simplifying the hardware, LPWA technologies are mainly designed to handle simple tasks. Therefore, there is no need to deploy expensive infrastructure. In other words, a single base station can connect a thousand end devices over a wide range. Hence, some additional parts, such as footprint and peak data rates, are reduced. Additionally, in order to further reduce the cost, most LPWA technologies used license-exempt bands such as ISM bands or TV-white spaces [1,4].

Finally, LPWA technologies must operate appropriately under the network growth. The efficient exploitation of the diversity in the channel, time, space, and hardware can help accommodate more connected devices. In addition, LPWA technologies employ multichannel to parallelize transmissions to and from the connected devices and make the communication resilient to interference. Moreover, LPWA networks need to resort to dense deployments of base stations. However, they must guarantee interference resistance among devices. So clearly, there is a trade-off between network scalability and keeping end devices low-cost and simple. In particular, LPWA technologies should scale to many connected devices and provide reliable and energy-efficient communication. So, it must adopt the most appropriate modulation schemes that help reduce collision and improve scalability. Unfortunately, most LPWA technologies adopt primarily uncoordinated and random schemas to access radio. Table 1 provides a general comparison between key LPWA technologies.

## 3. Related Work

### 3.1. Scalability in LPWA

Scalability is one of the main features and key design goals of LPWA networks. However, the current LPWA technologies’ scalability does not meet the future aspirations of IoT growth. In this section, we want to examine the research studies on LPWA technologies’ scalability to define the primary defects that limit these technologies’ scalability. Then, we explore scalability improvement-related work to show how the researchers address the scalability enhancement in LPWA technologies.

Lavric et al. [11] evaluated Sigfox scalability by studying the number of collisions and the Packet Error Rate (PER). Sigfox employs time and channel diversity techniques where every packet is transmitted through three different channels at different timeslots. This triple transmission is supposed to increase the network’s reliability. To assess the usefulness of the triple transmission feature, the authors conducted an extensive simulation study to compare it with the single transmission option. The results show that if the number of sensors is equal to or less than the number of communication channels, the three channels’ redundancy mechanism performs well. On the contrary, if the number of sensors is higher than the number of channels, the redundancy mechanism degenerates the performance of Sigfox networks. Generally speaking, Sigfox redundancy increases the collision probability and thus degrades the scalability.

According to the study conducted by Morin. et al. in [12], they found that the European Telecommunications Standards Institute (ETSI) regulation in the ISM band and the 1% duty cycle, along with the marketing limitation of Sigfox, limit the maximum capacity of Sigfox. Indeed, ETSI regulation restricts the number of continuous frames that nodes can transmit per hour, leading to a delay in some packets to the next hour. On the other hand, the Sigfox specifications recommend the node transmits only 140 packets per day. Therefore, the Sigfox redundancy mechanism, ETSI limitation, and Sigfox daily transmission specification lead to highly constrained scalability.

In the Slot-and Channel-Allocation protocol (SCAP) [13], the authors improved the scalability of Sigfox by using Time Division Multiple Access (TDMA) instead of Aloha as a medium access protocol while transmitting only on the orthogonal channels. Moreover, depending on the node’s geographical location and distance from the base station, they adopt time slot and channel autonomous allocation mechanisms on the nodes without communicating with the base station. Hence, the node can determine its own TDMA slot as well as its own channel identifier precisely without any extra packet exchange with the base station. Then, the packet will be transmitted only once instead of three times in the original Sigfox while guaranteeing its delivery to the base station, which will reduce the energy consumption and improve the throughput.

Still dealing with the scalability problem in Sigfox and other LPWA technologies, G.C. et al. [6] addressed the spectrum congestion in the Radio Resource Management (RRM) framework by taking advantage of the centralized nature of Software-Defined Networking (SDN) to assign the optimal channel for nodes. The SDN operates as a controller that collects sensed information according to the interference probability and stores it in a database. Then, based on the stored data, the appropriate subchannel is assigned for nodes, and the database is updated.

On the other hand, Pullmann and Macko [7] proposed a collision avoidance protocol Slot-Based Communication Planning Protocol (SCPP) that benefits from the exchanged message between the node and the access point. The protocol depends on the access point transition schedule. They mainly divided the communication time into planned and unplanned slots. The collision will be avoided by assigning planned slot time to only one node at a time. On the other hand, unplanned slots are assigned to any node where collisions may occur.

Regarding LoRa scalability, Lavric and Papa [14] estimate the maximum number of LoRa nodes that can communicate on a single channel based on the collision rate. They observed that the low transmission rate, duty cycle, and the number of nodes limited LoRa network’s performance. In particular, the low transmission rates increase the number of collisions as the used spreading factors increase the time on-air. Furthermore, the duty cycle parameter limited the number of transmitted packets, and many nodes resulted in a high collision.

Moreover, the scalability analysis of LoRa in [15] by Mikhaylov et al. showed that the single cell of LoRaWAN can serve millions of devices that send a few bits daily. Still, they should be near the access point, especially with high upload traffic nodes. Because the upload rate for distant nodes decreases as the distance between the nodes and the base station increases, this requires effective data rates management. Furthermore, the duty cycle restriction, the absence of any channel assessment mechanism on LoRa, and the use of acknowledgement affect LoRa’s scalability. In particular, the collision will increase the complexity of demodulating packets in dense networks.

Furthermore, Abeille et al. [16] studied the impact of confirmed vs unconfirmed messages and downstream traffic on LoRaWAN networks’ scalability through the ns-3 module. They found that the confirmed messages adversely affect the packet delivery ratio of upstream messages, where the packet is considered delivered if the node receives an acknowledgement. Dense access points can alleviate this effect where the downstream traffic spreads over the gateways. However, the duty cycle restrictions still limit the networks’ capacity.

Reynders et al. [8] utilized the concept of scheduled communication in order to enhance the scalability and reliability of LoRaWAN by proposing a new MAC layer protocol called RS-LoRa. RS-LoRa mainly operates through two scheduling steps. Firstly, the access point schedules nodes based on dynamic specification of each channel’s signal strength and spread factors. Secondly, the nodes, based on access point scheduling information, decide their transmission power, spreading factors, the selected channel for data transmission, and the appropriate time. Note that RS-LoRa mainly depends on downlink communication which is also subject to duty cycle constraint. Consequently, updating all nodes with appropriate transmission parameters is a time consuming task that depends on the network size.

Aggarwal and Nasipuri, in [9], also work to improve scalability in LoRa. They suggested an approach to reduce the number of collisions in LoRa networks by allocating multiple spreading factors in the same network zone. In particular, the studies indicate an optimum fraction for allocating two different spreading factors in the same zone, which increases the successful packet reception probability. Thus, they assign all nodes the smallest spreading factors and configure a fraction of the total devices to use a higher spreading factor, leading to a change in the collision domain that reduces the number of collisions.

Al Ahmadi et al. [17] benefit from the availability of several spreading factors to enhance the scalability of LoRa. They proposed an Annulus-based distribution algorithm, which divided the area around the access point into annulus cells and each cell into sub-cells. The partition process happens according to the spreading factors. In particular, LoRa supports six spreading factors (from 7 to 12). Accordingly, the network will be partitioned into six cells. For each cell, there is a set of eligible spreading factors based on the distance between the cell and the access point. Indeed, the closest cell has all six eligible spreading factors in contrast to the farthest cell with only one eligible spreading factor (12). Depending on these eligible spreading factors, each cell is further divided into subcells. Additionally, they assign a unique channel with specific transmission power for each cell. Thus, there will be no collision even if two nodes are simultaneously communicating on the same channel, thanks to the spreading factors’ orthogonality.

To summarize, the duty cycle regulation on the ISM band highly compromises the scalability of the LPWA technologies, namely Sigfox and LoRa. Regarding Sigfox, in addition to the duty cycle constraint, the Sigfox specifications and Sigfox redundancy based transmission mechanism further restrict the scalability of Sigfox networks. Similarly, the data rate management mechanism and the downlink traffic compromise LoRa scalability in addition to the duty cycle regulation. Many works appeared to address the scalability in LPWA technologies by utilizing different mechanisms such as time slots and spreading factors’ optimal distribution. However, these works succeeded with a limited improvement of the LPWA scalability. Still, the ISM band duty cycle restriction highly compromises the LPWA scalability. Therefore, an LPWA technology with no duty cycle restriction would be a better candidate for a scalable IoT network. Ingenu-RPMA is an LPWA technology that works on the 2.4 GHz band and implements the DSSS signal, where there are no imposed global limitations [10].

### 3.2. Scalability in RPMA

Scalability is considered one of the most critical design goals of LPWAN communication models. In addition, it represents one of the main requirements of IoT connectivity. Many studies in the literature address the performance of LPWA technologies. In this section, we explore some of these studies which address RPMA performance. Unfortunately, there are no sufficient and comprehensive studies on RPMA. However, some researchers study RPMA as part of their work.

Naik [18] examines RPMA as a spread spectrum technology, which is considered resistant to interference, where any jamming signals will be rejected and cannot be de-spread. However, it suffers from additional interferences, e.g., self-noise, which happens in dense environments. Mainly the node becomes an interference source for other nodes within the same channel. Moreover, increasing the number of nodes will increase the self-noise (interference), restricting the network capacity and scalability. Additionally, other interference can be generated by other technologies that operate within the same band (bad-neighbor).

According to the studies in [19,20], operating in the 2.4 GHz ISM band provides a broad, flexible spectrum without suffering from the duty cycle constraint such as in the sub–GHz ISM band. Consequently, it offers a higher network throughput and capacity. Moreover, both studies indicate that operating in the 2.4 GHz ISM band is exposed to interference from other technologies working in this band which may limit the signals coverage.

Similarly, Ikpehai et al. [21] point out that working in greater than 2 GHz bands suffers from additional path loss depending on the nature of the obstacles in the network domain. Indeed, they noticed that the ability to overcome obstacles will decrease as frequency increases and hence it will affect the network coverage. So, to enhance RPMA coverage, they increased the receiver power by 10%, thus improving RPMA coverage by 135%. Again in [22], the authors indicate that operating on the 2.4 GHz ISM band imposes interference with Wi-Fi and Bluetooth, which increases the propagation loss. However, RPMA has a good architecture which gives RPMA a better capacity than the other competing technologies.

Most of these studies focus on the 2.4 GHz ISM band and the effect of operating in this band on RPMA performance. However, to the best of our knowledge, there is no work in the literature that addresses the impact of the technical aspects of RPMA on the extensibility of RPMA. In this paper, we shed light on the influence of spreading factors on the scalability of RPMA through mathematical analysis and simulation analysis. We discussed the benefit of the multichannel communication scheme over RPMA capabilities. Also, we explore the effect of adding the random delay before every transmission on RPMA capacity and scalability.

## 4. RPMA Background

Random Phase Multiple Access (RPMA) is an LPWAN technology patented by Ingenu [23]. RPMA is designed to provide the optimal connection for billions of connected IoT devices requiring low cost and high capabilities. RPMA has many advantages compared to other LPWA technologies, but the most significant is RPMA’s ability to achieve the highest coverage and capacity [24]. RPMA works on the 2.4 GHz ISM band, unlike most other LPWA technologies that work on the sub-GHz ISM band. In particular, the 2.4 GHz ISM band is not only an unlicensed free-spectrum of a wide 80 MHz [2] width but also, it is a global band. Moreover, there are no imposed duty cycle limitation in the 2.4 GHz ISM band on the DSSS, which is the air interface used in RPMA physical layer [10,24].

DSSS plays the primary role in providing RPMA with high capacity. In particular, DSSS has multiple features that make it suitable for LPWA technologies. Indeed, the processing gain, which is the amount of used spreading highly impacts the coverage. In fact, more processing gain means more receiver sensitivity and then more reliable coverage. On the other hand, more processing gain means a lower data rate, which can be tolerated as LPWA IoT devices require a low data rate even lower than the voice rate. Thus, RPMA increases the amount of processing gain to increase the spreading. RPMA has a processing gain of 39 dB or 8192 chips per coding symbol. Another notable point is that DSSS is considered resistant against interference due to pseudo noise characteristics. In particular, the transmitted signals look like noise to other users, even within the same frequency. Consequently, RPMA can demodulate more than 1000 simultaneously overlapping signals [10].

RPMA operates in the 2.4 GHz band of width 80 MHz. The RPMA waveform has a 1 MHz bandwidth. Thus, each RPMA channel has a 1 MHz bandwidth separated by 2 MHz as a guard band resulting in 40 channels available for RPMA in the 2.4 GHz ISM band. To avoid interference with Wi-Fi, RPMA’s primary channels are defined where Wi-Fi is not typically deployed. However, RPMA assumes a single shared channel can be used for the whole network [24].

RPMA divides this 1 MHz channel into slots or frame. Indeed, RPMA has a long frame measured in seconds instead of milliseconds, similar to most of LPWA technologies [10]. RPMA frame structure is based on Time-Division Duplex Approach (TDD) that is characterized by a phenomenon known as “channel reciprocity”; where the uplink frequency is the same as the downlink frequency. Along with TDD, RPMA used an open loop power control, where the end node determines the uplink transmission power by measuring the downlink receiver power. In particular, when the uplink channel condition is faulty, RPMA will increase the spreading and transmission energy and decrease them if the channel conditions are good. Thus, RPMA dynamically improves the capacity, scalability, and battery lifetime whenever possible [24].

**Figure 1:**
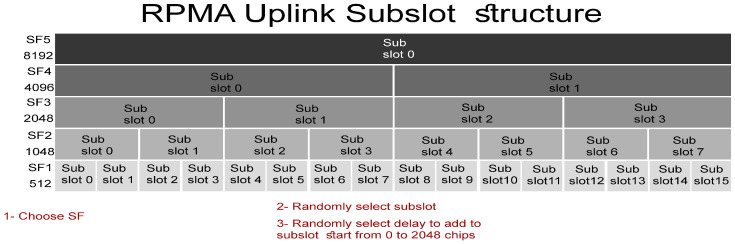
Uplink subslot structure [10].

**Figure 2:**
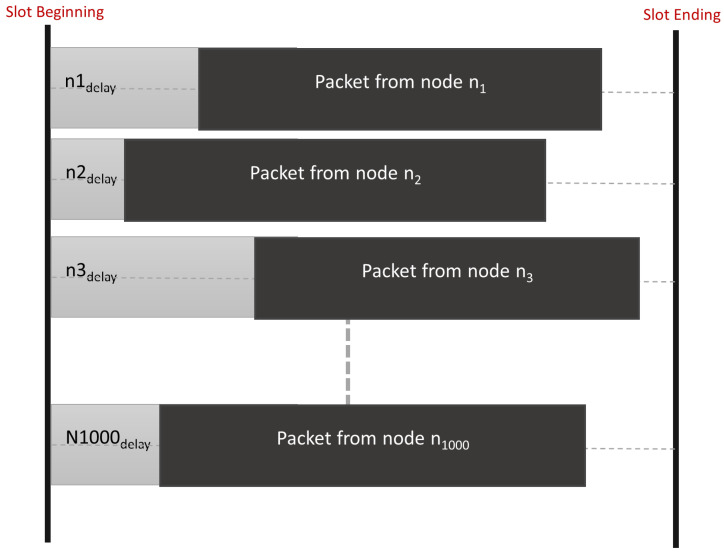
RPMA scheme [10].

The RPMA uplink slot is divided into subslots where the total number of subslots depends on the used SF [4], as shown in Figure 1. Indeed, RPMA uses five SFs: 512, 1024, 2048, 4096, and 8192 [10]. For instance, the total number of subslots for SF 512 is 16 while it equals 8 for SF 1024. Hence, if a node uses SF 512, it has to choose among 16 available subslots in order to send its message. In other words, if the node wants to send a message, it first needs to determine the proper SF based on the strength of the received signals from the Access point. Second, the node needs to randomly choose which Sub-slot to use. After that, the node must randomly choose an intentional delay from 0 to 2048 chips as described in Figure 2 [24]. Note that this intentional delay will help reduce packet collisions at the access point. Indeed, the RPMA access point can successfully demodulate partially overlapping messages as long as they do not arrive at the exact moment. It is worth pointing out that in RPMA, each node is identified by a unique gold code where the low auto correlation features of gold code coupled with unsynchronized transmission allowed correct decoding [25].

### RPMA in the Real World

RPMA operates in the global 2.4 GHz band. Consequently, RPMA networks are easily deployed globally without any restrictions. At Mobile World Congress (Barcelona 2017), Ingenu announced that RPMA networks are extended to 29 countries (such as Japan, Aruba, China, Gulf Cooperation Council (GCC), Chile, Australia, Thailand, Canada, USA, Taiwan, Brazil, South Africa, Nigeria, etc.) [26]. In particular, RPMA has a prominent use in monitoring oil and gas. Therefore, Ingenu cooperates with expert companies to deploy RPMA networks like in the US and Nigeria [27,28]. In addition, RPMA provides intelligent cities with agriculture, logistics and automotive services to people where it is deployed, such as in Brazil [29]. In this context, some works in the literature provide smart city planning based on RPMA networks. For example, in [30,31,32], the authors leverage RPMA’s capacity and coverage to introduce smart city planning in Batam Island with an area of 1595 km^2^, Bandung city covering 167.3 km^2^ and Surabaya city with a surface of 350.5 km^2^ for Advanced Metering Infrastructure (AMI) Services, respectively. The planning aims at determining the minim number of access points to provide sufficient capacity and coverage through the use of a Forsk Atoll simulator and an Erceg-Greenstein Propagation Model. Simulation results can be listed as follows: Batam Island requires 23 access points with 69.21 dBm as the average receiving signal level. Bandung city demands 12 access points; the average best signal level is −59.43 dBm. Finally, Surabaya required 21 RPMA access points at a signal level of −72.02 dBm.

## 5. Mathematical Analysis

This section introduces a mathematical analysis for RPMA scalability based on collision probability. In particular, we consider that a collision happens if two or more messages are sent simultaneously in the same subslot using the same SF on the same uplink channel. It is worth noting that, in order to assess the contribution of the intentional delay in RPMA transmission, we will start by assessing the probability of collision without considering it. As a second stage, we will consider the intentional delay and assess the achieved reduction in the probability of collisions.

RPMA has five Spreading Factors (SFs) which are (512, 1024, 2048, 4096, and 8192); for short, we will call them, hereafter, SF1, SF2, SF3, SF4, and SF5, respectively. Accordingly, the total number of transmission subslots based on the used SF equals:

(1) $$Subslot_{SF}=\frac{8192}{SF}.$$

As a result, the lower the used SF, the more the available subslots and hence the lower the collision probability. To illustrate this, SF1 has sixteen subslots, consequently the lowest collision probability, while SF5 has only one subslot, thus the highest collision probability since nodes that choose SF5 will use the same subslot for transmission and hence will inevitably collide if they are transmitting on the same channel. Therefore, we derive the collision probability at each SF separately on a given channel. First, let us assume that $N_{t}ot$ is the total number of nodes in the network. If we suppose that the total number of available RPMA channels is $N_{ch}$ that are randomly chosen by each node, then on average, the number of nodes on a given channel $N_{nodes\_ch}$ is:

(2) $$N_{nodes\_ch}=\frac{N_{tot}}{N_{ch}}.$$

Moreover, opting for a random choice of a spreading factor will reduce the average number of nodes that uses the same SF on the same channel to $N_{nodes\_ch\_SF}$

(3) $$N_{nodes\_ch\_SF}=\frac{N_{nodes\_ch}}{5}.$$

Note that, among these $N_{nodes\_ch\_SF}$ nodes, only the ones that select the same subslot will collide. To find the probability of collision per SF, let us define $P_{a_{slot}}$ as the probability of accessing a given slot (RPMA frame) by a given node. For the proportion of nodes that use a given channel with a given SF, a successful transmission is achieved only if a unique node from these ones access any subslot from the available ones (subslots_SF) for that SF while the others do not. Note that subslots_SF = 16 for SF1. Accordingly, the probability of successful transmission can be expressed as follows:

(4) $$P_{success\_CH\_SF}=\sum_{i=0}^{subslots\_SF}i{\left(P_{a_{slot}}\right)}^{i}{\left(1-P_{a_{slot}}\right)}_{nodes_{CH}\_SF}^{N}-i$$

(5) $$P_{col_{\_}SF}=1-P_{success\_CH\_SF}=1-\sum_{i=0}^{subslots\_SF}i{\left(P_{a_{slot}}\right)}^{i}{\left(1-P_{a_{slot}}\right)}_{nodes_{CH}\_SF}^{N}-i.$$

For the RPMA-Single channel, it is straightforward for any SFs that, if the number of nodes choosing the same SF is more than the number of possible sub-slots, 100% collision happens at any subslot. For instance, SF1 divides the slot into 16 subslots. Thus, the maximum number of nodes transmitting in this slot using SF1 is only 16, and similarly for the other SFs. Consequently, the maximum number of nodes that can successfully send at a given slot is only 31 (16 (SF1) + 8 (SF2) + 4(SF3) + 2(SF4) + 1(SF5)) considering all the available SFs. To put it in another way, if there are 1000 nodes in the network, the average number of nodes using a given SF is $N_{nodes\_ch\_SF}$ = 1000/5 = 200. With high $P_{a_{slot}}$, the probability of collision will be so high as a maximum of 16 subslots are available for nodes using SF1.

**Figure 3:**
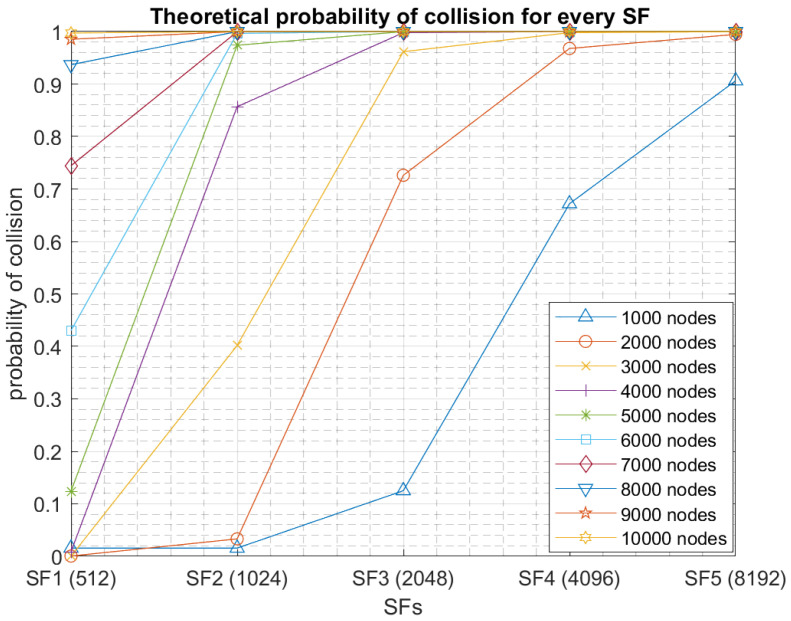
The theoretical probability of collision for every SF.

On the other hand, by using the multichannel feature in RPMA, the collision probability is highly reduced. Specifically, for RPMA, there are 40 channels in the 2.4 GHz ISM band. However, two channels are reserved for downlink communication. So there are 38 channels available for RPMA uplink communication. Consequently, considering the same example of 1000 nodes, $N_{nodes\_ch\_SF}$ = 1000/(38 × 5) = 5.26. Only 6 nodes, in average, are competing for the subslots of any SF. Thus, if the access probability is high, then the probability of collision is also high for SF5, SF4 and SF3 as the number of available subslots is less than 6. Figure 3 shows the theoretical probability of collision for every SF when the network size is varying from 1000 to 10,000. In our work, we suppose that nodes randomly choose the channel with a probability of 1/38. For example, if 100 nodes use SF1 on average, only three nodes are transmitting at every channel, coupled with 16 subslots for SF1; consequently, the probability of collision is almost non-existent or equal to zero. However, if these nodes use SF5, the collision probability is still high. We observe that the transmissions using high SFs will increase the collision probability. However, utilizing multichannel will improve the whole network capacity.

**Figure 4:**
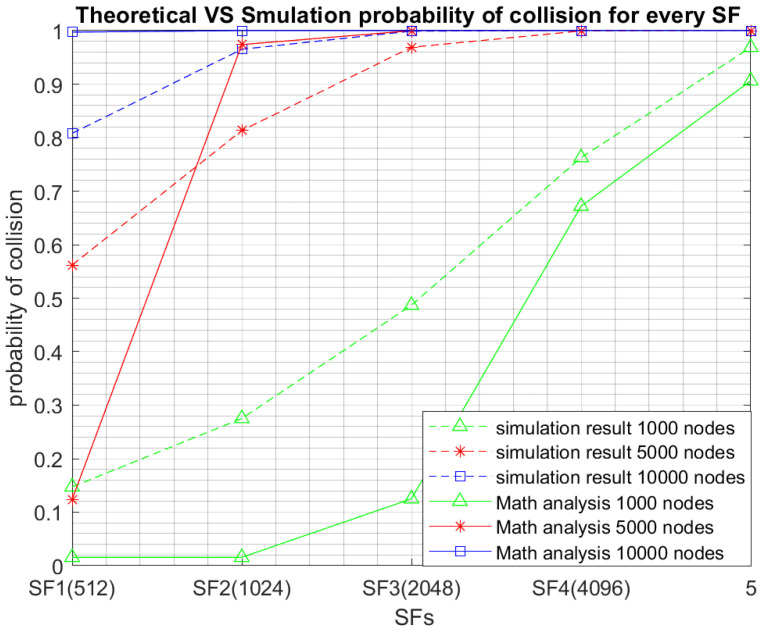
Theoretical collision probability versus simulation based collision probability.

Figure 4 displays the probability of collision for every SF by using both the theoretical analysis and the simulation. For comparison purposes the results relative to three network sizes are depicted in Figure 4. Although being overall close, we notice that the difference between the simulation and theoretical results is larger for small SFs and is considerably reduced for higher SFs. The main rational behind this is the relationship between the selected SF and the number of available subslots which will impact the accuracy of our theoretical model. Indeed, for instance for SF1, 16 subslots are available. According to our theoretical model, we assume that if at maximum 16 nodes access a given channel using SF1, no collision will happen which is not totally accurate, as collisions still may happen if two nodes among the 16, choose the same subslot. This assumption explains the larger probabilities of collision achieved by simulation as more collision scenarios may happen. Note that we adopt this assumption in our theoretical model to simply the model and, most importantly, the impact of our assumption will fade with higher SFs. In fact, the greater the selected SF, the smaller the number of available subslots and hence the number of collision scenarios when the number of accessing nodes equals the number of subslots will be reduced and hence very close results are achieved by both the simulation and theoretical analysis. Indeed, for SF5, one slot is available for transmission. According to our theoretical analysis, if two or more access the same channel using SF5, a collision will inevitably happen, which is the exact accurate scenario.

## 6. Simulation Analysis of RPMA

In this section, we study RPMA characteristics and discuss the scalability of RPMA. We simulated RPMA behavior using MATLAB based on RPMA attributes granted from [10]. Firstly, we deeply investigate the relationship between the available SFs and the associated number of subslots and their impact on the network performance while ignoring the intentional delay before any transmission. Indeed, as in RPMA, the number of available subslots depends on the selected SF; we want to investigate the effect of such a relationship on the network scalability. Considering the intentional delay characteristic before transmission will prohibit shedding light on the dependency relationship between the selected SF and the number of available subslots. Then, in a second stage, we investigate the performance improvement in RPMA achieved by adding the intentional delay feature and explore its impact on RPMA scalability.

### 6.1. Simulation Analysis of the Original RPMA Frame

We investigate the effect of using the available SFs on the collision probability, PDR (Packet Delivery Ratio) and the network throughput. At this stage, we simulated 1000 nodes transmitting in one day (86,400 seconds). First, we set all nodes to use only one SF (SF1), then increased to two SFs (SF1 and SF2) and so on. Furthermore, to assess the effect of multichannel communication schema, we repeated these simulations using several channels, namely 10, 20, 30, and 38. First, it is important to point out that, other than expected, the collision probability is not minimized when using all the available SFs. Indeed, we expected that the collision probability would decrease with the increased number of used SFs as nodes transmissions have more chances to be separated using large number of SFs thanks to the SFs orthogonality. However, using all available SFs is not always beneficial, especially when the nodes are within the same distance from the access point. The relationship between the used SF and the number of available subslots will restrict the optimal number of used SFs in the network. Recall that, according to RPMA specifications, the slot will be divided into subslots according to the used SF using Equation (1).

**Figure 5:**
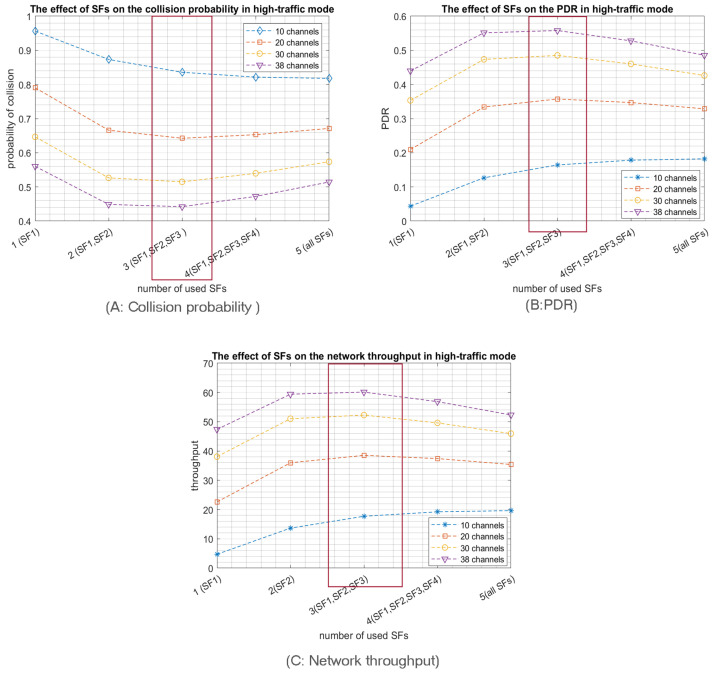
The effect of the number of used SFs on the network performance for 1000 transmitting nodes in high-traffic mode.

**Figure 6:**
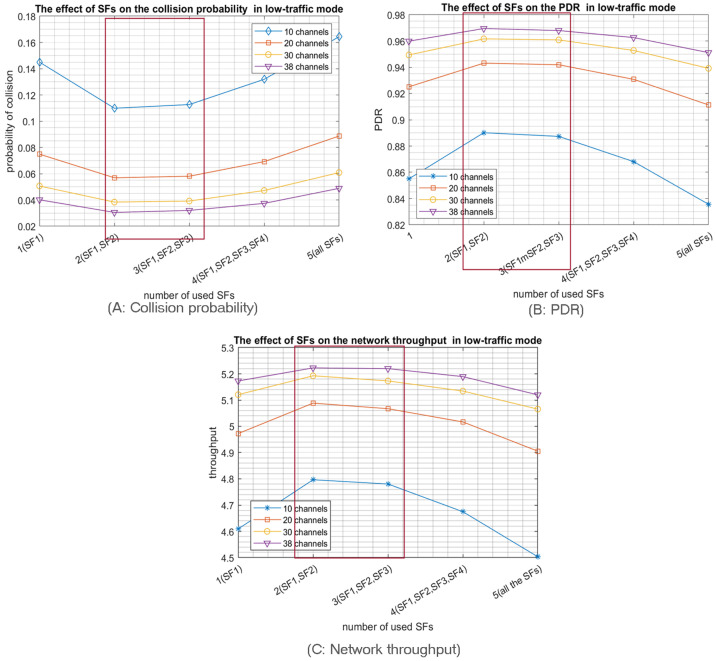
The effect of the number of used SFs on the network performance for 1000 transmitting nodes in low-traffic mode.

Accordingly, low SFs have more subslots and, thus, more choices for nodes to select a sub-slot for transmission. For instance, by using SF1 “512”, the slot will be divided into sixteen subslots; thus, the node has sixteen options to select from to transmit. In contrast, using SF5 “8192” consumes all the slot, i.e., no options since the nodes selecting SF5 will have only one subslot at their disposal for transmission; hence collision is inevitable. Similarly, and according to our mathematical modelling of the probability of collisions, nodes using SF4 have only two subslots as options. Therefore, the probability of collision equals (0.65) in high-traffic mode by using 20 channels. According to Figure 5A. when the nodes are relatively close to the access point, the optimal number of used SFs is the first three SFs, especially when the access probability Pa is high, resulting in high-traffic mode. However, in IoT LPWA, traffic usually tends to be low (low-traffic mode), so similarly, adopting the first three SFs or the first two SFs achieves the same minimum collision probability, as shown in Figure 6A.

The collision probability results indicate that using only the 3 lower SFs for the high-traffic mode and the 2 lower SFs for the low-traffic mode produce the best PDR and network throughput as both configurations achieve the lowest collision probabilities. For example, Figure 5B shows that the best PDR was obtained using only the three Lower SFs in high-traffic mode for various number of channels while in Figure 6B, the best PDR was obtained using only the two Lower SFs in the low-traffic mode. Consequently, the network throughput will be enhanced, as shown in Figure 5C. Note that, similar to the PDR results, the best throughput is reached by using only the three lower SFs for high-traffic mode while in low-traffic mode, the best throughput is achieved by using the two lower SFs, as shown in Figure 6C. Consequently, when the coverage range of the RPMA access point is relatively small, it is highly preferred for the nodes to choose only among the first three SFs to proceed for transmission. It is worth pointing out that the simulation results confirm our mathematical results, as shown in Figure 4.

As a second main expected result, opting for a multichannel communication scheme will highly reduce the collision probability, as shown in Figure 5A and Figure 6A. In particular, RPMA has 40 channels in the 2.4 GHz ISM band band, where two are reserved for downlink communication; thus, there are 38 channels available for uplink communications that can be utilized to enhance RPMA scalability. For example, Figure 6A shows how the collision probability when the number of selected SFs equals two is reduced from 0.11 in 10 channels scenario to 0.03 in 38 channels scenario when the traffic mode is low. Similarly, the PDR is increased from 0.89 when using 2 SFs in 10 channels scenario to 0.97 in 38 channels scenario, as shown in Figure 6B. As a result, the network’s throughput is improved from 4.8 packet/s in 10 channels scenario to 5.2 packet/s in 38 channels scenario, as shown in Figure 6C.

In summary, using only the two or three lowest SFs while employing a multichannel communication schema highly reduces the collision probability in the IoT RPMA-based communication model.

**Figure 7:**
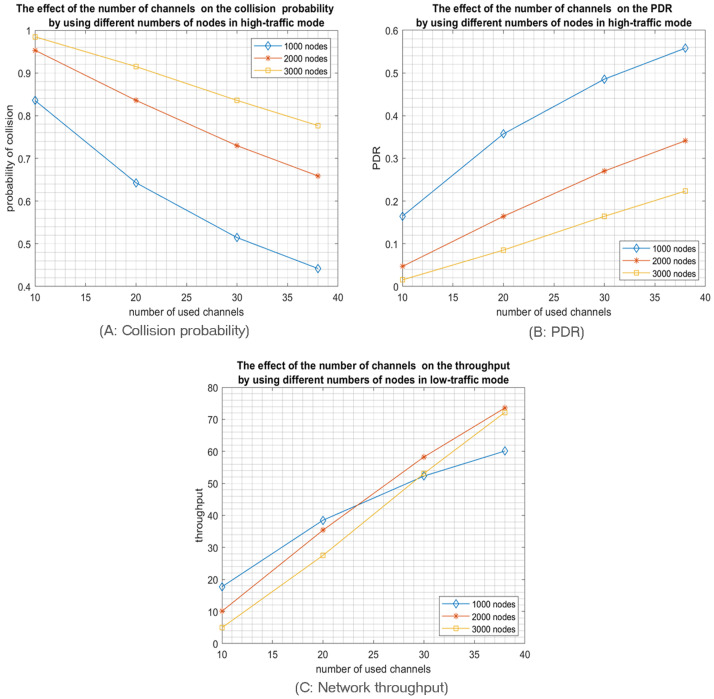
The effect of the number of channels on the network performance for different network sizes operating in high-traffic mode and using only the first 3 SFs.

**Figure 8:**
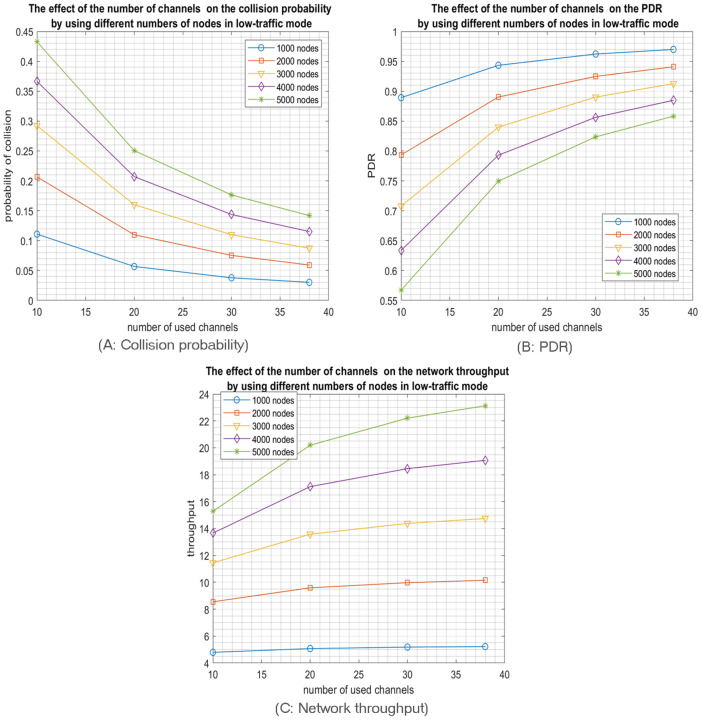
The effect of the number of channels on the network performance for different network sizes operating in low-traffic mode and using only the first 2 SFs.

**Figure 9:**
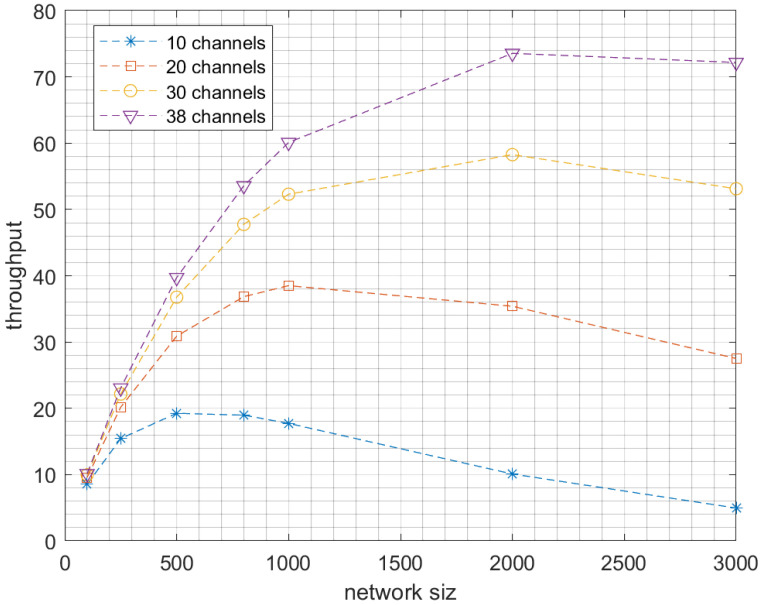
The effect of network size on network’s throughput by using different number of channels.

Now, we investigate the impact of increasing the network size in a multichannel communication model while using the optimal number of SFs, namely two SFs for low-traffic mode and three SFs for high-traffic mode. So, we tested different numbers of nodes, from 1000 up to 5000 nodes. As expected, for both scenarios—high and low-traffic modes—increasing the network size will increase the collision probability which will reduce the packet delivery ratio but increase the network throughput as shown in Figure 7 and Figure 8. Indeed, the network throughput is expressed as the number of successfully received packets per unit of time. Despite the increase of the collision probability in larger network sizes, the network will succeed in delivering more packets as more nodes are sending. However, this increase will certainly reach a maximum when the network is saturated where the throughput will start decreasing as the collision impact will take over the traffic rate as shown in Figure 9. Please note that such behavior is easier to observe in the high-traffic mode as the network may reach the saturation faster. However, in low-traffic mode shown in Figure 8C, the network may never reach the saturation that’s why only the first part of the throughput behavior will be experienced where the throughput is increasing with the increase of the number of nodes since more nodes are sending.

**Figure 10:**
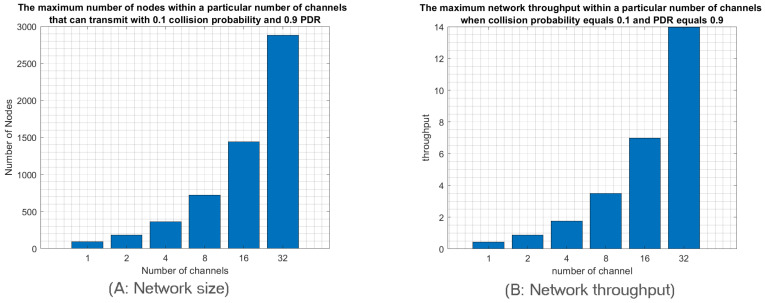
The network size and throughpu t within a particular number of channels that can transmit with 0.1 collision probability and 0.9 PDR in low-traffic mode.

Finally, we study the relationship between the network size and the number of deployed channels to achieve idealistic IoT networks. In other words, we will investigate how many nodes can transmit through a particular number of channels to achieve an idealistic network that the IoT application requirements may impose. We suppose that an idealistic network is characterized by a collision probability around 0.1 and PDR around 0.9. Since IoT applications produce low traffic, we will focus here only on low-traffic mode. According to Figure 10, we notice that idealistic network specification when a single channel is used can be achieved with a network size of 90 nodes. However, 32 channels can support a network size of 2800 nodes while achieving the required probability of collision and PDR, namely 0.1 and 0.9, respectively.

We observe a linear relationship between the number of used channels and the number of deployed nodes to achieve the idealistic IoT network requirements. Indeed, adding one more channel will increase the number of deployed nodes by the same amount as shown in Figure 10A. Therefore, employing all 38 uplink channels will enhance RPMA scalability 38 times compared to the single channel scenario. Moreover, it is worth pointing out that adopting n channels will also improve the throughput n times, as shown in Figure 10B. Therefore, using 38 channels will improve the network throughput 38 times.

### 6.2. Simulation Analysis of RPMA Frame with Intentional Delay

One of the essential distinguishing features of RPMA from the other LPWA technologies is the ability to demodulate overlapping signals due to the properties of DSSS using different spreading factors. Furthermore, as we mentioned in Section 3, each node adds an intentional delay before proceeding transmitting, and hence the access point can further demodulate overlapping signals even using the same SF as long as their arrival time is different [10,24,25].

In this section, we investigate the performance enhancement that may be achieved by considering the intentional delay feature. Accordingly, each node chooses an intentional delay from (0 to 2048 chips or [0 to 2.048 × 10^−3^ second]) before starting transmitting [10] as shown in Figure 2. Thus we adjust our simulator to consider the arrival time to calculate the collision probability, PDR and network throughput. Accordingly, two nodes that use the same channel with the same SF and select the same subslot will not collide if they have a different arrival time to the access point.

**Figure 11:**
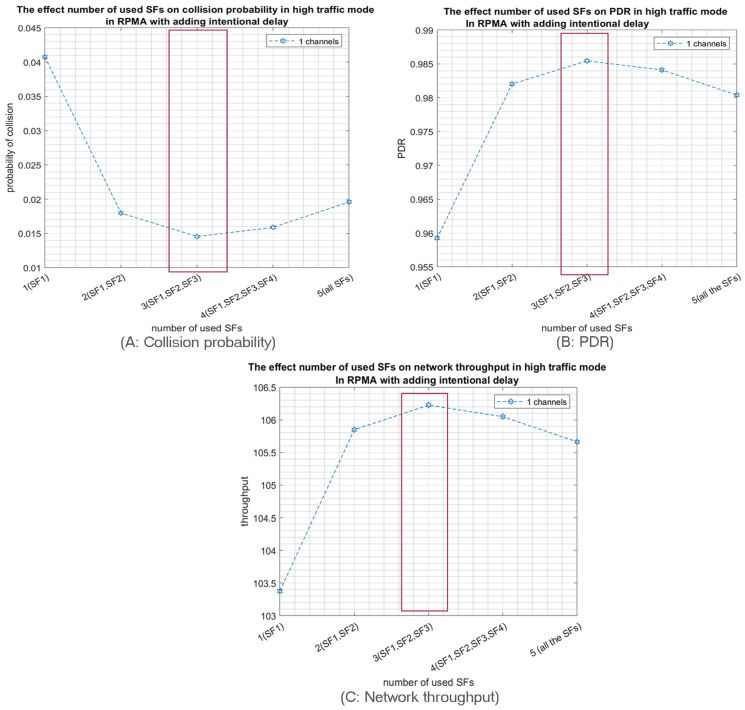
The effect of the number of used SFs on the network performance when 1000 nodes are transmitting in the high-traffic mode in only one channel with intentional delay.

**Figure 12:**
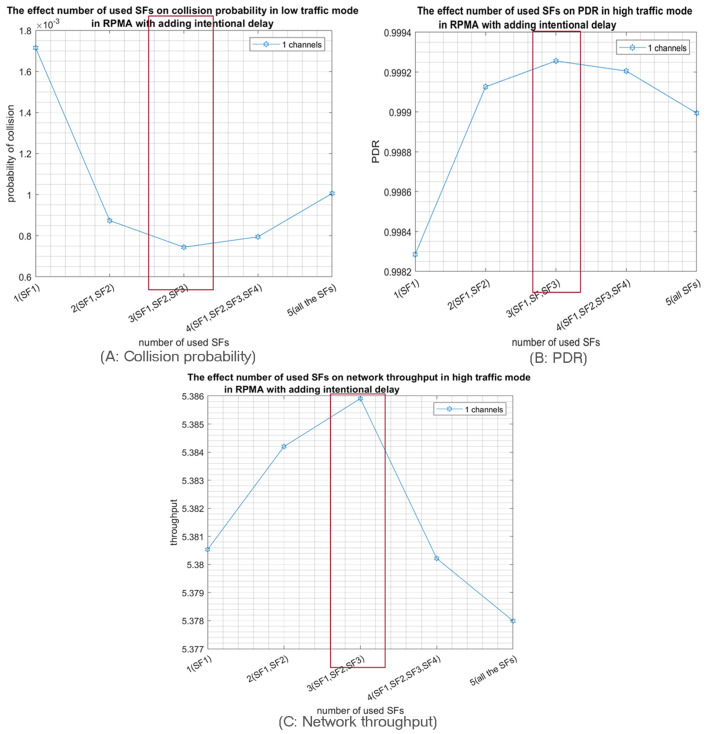
The effect of the number of used SFs on the network performance when 1000 nodes are transmitting in the low-traffic mode in only one channel with intentional delay.

First, we study the probability of collision as function of the number of used SFs when 1000 nodes are sharing a unique channel. Noticeably, the collision probability is highly declined for both high- and low-traffic modes, as shown in Figure 11A and Figure 12A respectively. Consequently, the PDR and the network throughput are both improved as shown in Figure 11B,C for high traffic and in Figure 12B,C for low traffic. Likewise, using only the three first SFs is the optimal solution in this case.

**Figure 13:**
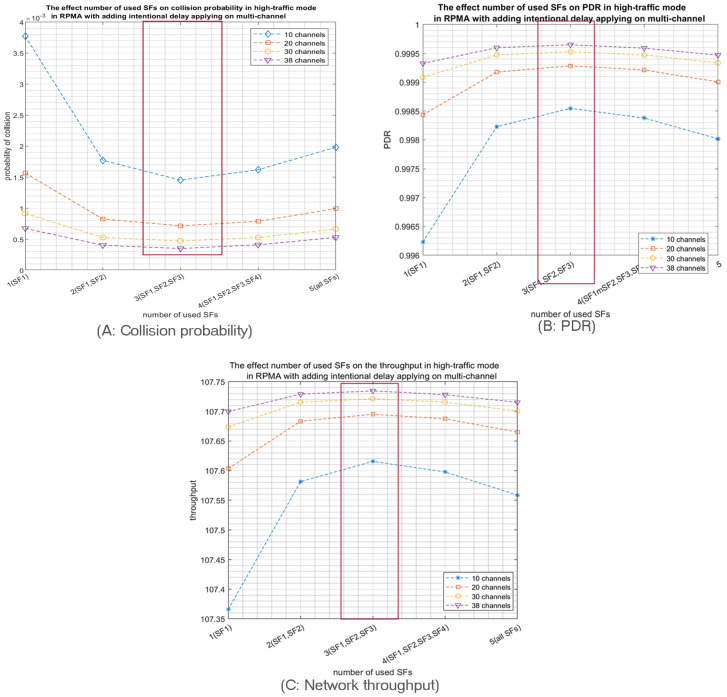
The effect of the number of used SFs on the network performance when 1000 nodes are transmitting in the high-traffic mode with intentional delay by using a different number of channels.

**Figure 14:**
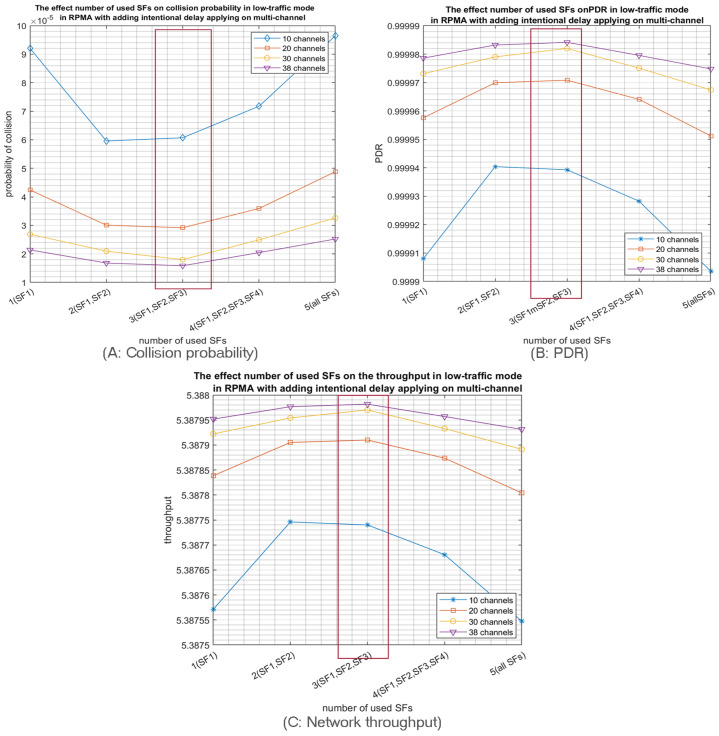
The effect of the number of used SFs on the network performance when 1000 nodes are transmitting in the low-traffic mode with intentional delay by using different numbers of channels.

Then, we increased the number of used channels to test the impact of multichannel on RPMA scalability. Indeed, as expected, using multiple channels (10, 20, 30, 38), highly decreases the collision probability for both high and low-traffic modes. For example, when 1000 nodes are transmitting in high-traffic mode, the achieved collision probability in 10 channels schema equals (3.7 × 10^−3^) by adopting the first three SFs as shown in Figure 13A. Most importantly, this probability is decreased to (5 × 10^−4^) by using 38 channels. Similarly, the PDR is increased from (0.9965) in 10 channels scenario to (0.9995) in 38 channels scenario as depicted in Figure 13B. Moreover, Figure 13C shows the achieved improvement in the network throughput with the increase of the number of used channels. Similar results are accomplished when 1000 nodes are deployed in low-traffic mode. Once again, the multichannel demonstrates significant improvement in network efficiency in terms of collision probability, PDR and network throughput, as shown in Figure 14. Furthermore, as depicted in Figure 13A and Figure 14A, adopting the first three SFs produced the lowest collision probability for both high- and low-traffic modes.

**Figure 15:**
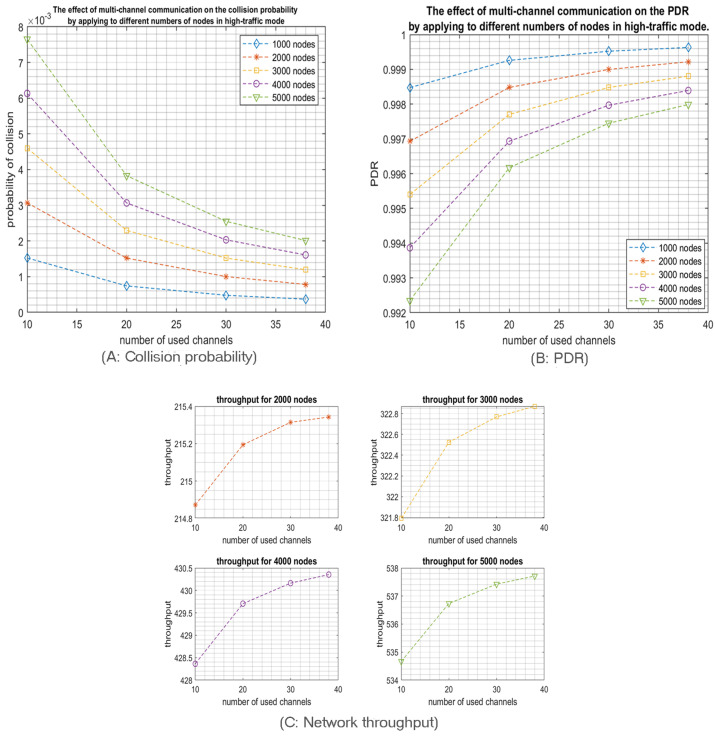
The effect of the number of channels on the network performance for different network sizes operating in high-traffic mode and using only the first 3 SFs with intentional delay by using different numbers of channels.

**Figure 16:**
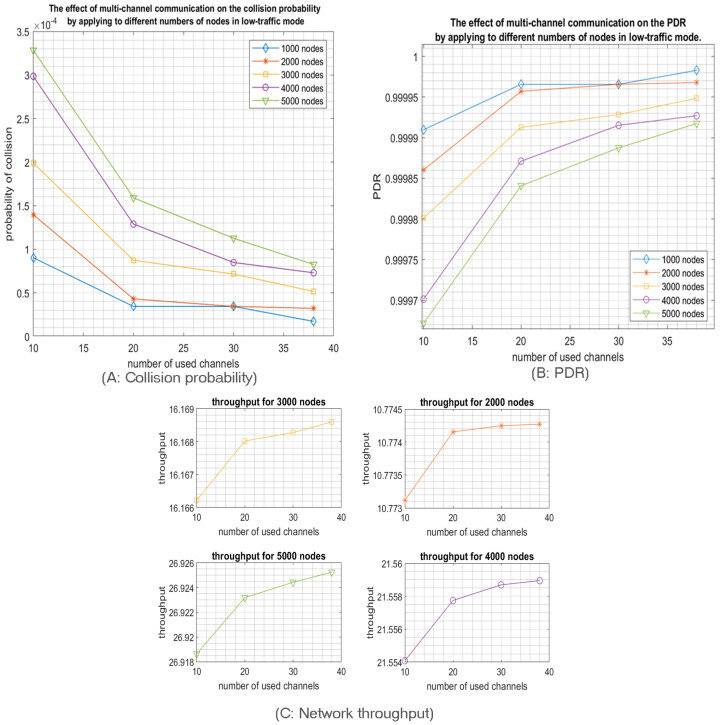
The effect of the number of channels on the network performance for different network sizes operating in low-traffic mode and using only the first 3 SFs with intentional delay by using different numbers of channels.

Now, we increased the number of nodes to explore the impact of multichannel communication schema on the collision probability. As expected, the collision probability increases by increasing the number of nodes in the network, as shown in Figure 15A and Figure 16A. However, opting for the multichannel communication scheme will highly reduce the collision probability. Then, we tried different numbers of nodes to explore the impact of multichannel communication schema on the network transmissions by adopting only three SFs (SF1, SF2, SF3), which gives the best collision probability.

Firstly, we simulated different numbers of nodes ranging from 1000 to 5000 that transmit in high-traffic mode. As expected, and explained previously, the collision probability is increasing with the increase of the network size which will cause the PDR to decrease. However, despite the increase in the probability of collision, the network throughput will increase with the network size as more traffic is generated and the collision impact is minimized. Here again, opting for the multichannel communication scheme will highly reduce the collision probability. For example, by using 5000 nodes sharing 10 channels, the collision probability equals (7.7 × 10^−3^); in contrast, it is only (2 × 10^−3^) when using 38 channels. Thus, the PDR increases from (0.9923) up to (0.9980). Moreover, the network throughput is improved from (534.67) to (537.71) packet/s as shown in Figure 15.

Again, we re-tested the effect of using different numbers of nodes ranging from (1000 to 5000) but in low-traffic mode. Figure 16 demonstrated that utilizing multichannels helps reducing the collision probability which will increase the PDR, and hence will highly enhance the network throughput.

**Figure 17:**
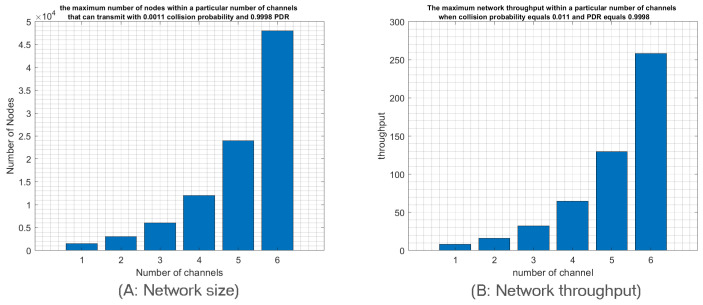
The network size and throughput with a particular number of channels that can transmit with 0.01 collision probability and 0.99 PDR in high-traffic mode.

It is clear that RPMA has vast scalability with the multichannel feature. So, what is the largest network size that will satisfy the requirements of an idealistic RPMA networks when the multichannels communication scheme is adopted. Considering the delayed chips feature, more stringent requirements can be imposed. We assume that the IoT application goal is to achieve a collision probability around 0.0011 and a PDR of around 0.9998 to investigate the maximum number of nodes that can satisfy this requirement for every number of used channels. In low-traffic mode, note that when a single channel is used, this target collision probability is achieved with 1500 nodes. Similar to the previous simulation result, the number of nodes will be increased by n times with n channels as shown in Figure 17A. As a result, for 38 channels, 57,000 nodes will be transmitting with only a 0.0011 collision probability, which denotes the high scalability of RPMA networks. Furthermore, the network throughput will be increased by increasing the number of nodes, as shown in Figure 17B.

**Figure 18:**
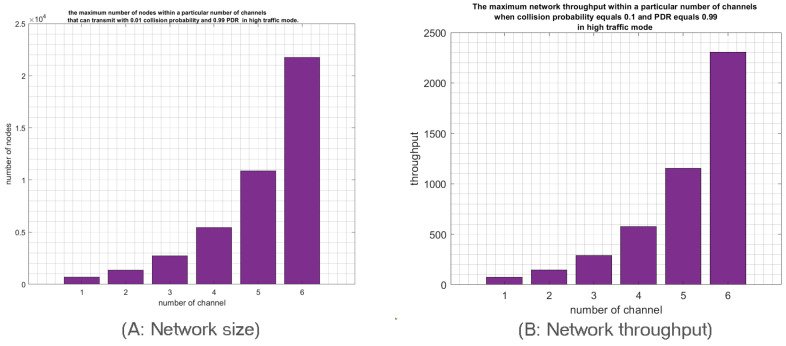
The network size and throughput within a particular number of channels that can transmit with 0.01 collision probability and 0.99 PDR in high-traffic mode.

Although the traffic in IoT tends to be low, especially when we talk about LPWA networks, we estimated the RPMA networks’ capacity in a high-traffic mode, which may help future studies to enhance RPMA scalability. Mainly, we supposed that idealistic RPMA networks in high-traffic mode have a collision probability should be around 0.01 and a PDR of about 0.99. We found that the target collision probability, and PDR are achieved when only 680 nodes are sharing a single channel. The same linear relationship holds also for the high-traffic mode. As a result, for 38 channels, 25,650 nodes will succeed in transmitting while satisfying the requirements. Likewise, the multichannel scheme helps to increase the throughput by n times compared to the single channel scenario, where n represents the number of channels, as shown in Figure 18. Finally, we conclude that the ability of RPMA to demodulate overlapped signals significantly improves RPMA scalability along with utilizing the multichannel feature. Consequently, multichannel RPMA can be considered a practical solution to satisfy the continuously increasing requirements for IoT applications.

## 7. The Impact of SFs Distribution on RPMA Scalability by Using Multichannel

Our previous work focused on only the first three SFs because it provides the minimum collision probability. However, in some cases, transmitting using high spreading power is necessary otherwise packet loss may be experienced. Accordingly, to preserve the transmission efficiency and mitigate collisions produced using the higher SFs, we proposed three SFs distribution algorithms especially when the IoT network span a large geographical area. Moreover, we study the impact of these algorithms on PER (packet error rate), the PDR and the throughput.

### 7.1. Identifying the Coverage Range for Every SF

Before evaluating the impact of SF distribution on RPMA scalability, the coverage of every SF must be determined for appropriate distribution.

Based on [33], the noise level of RPMA is:

(6) N=−174+NF+10logB,

where −174 is the thermal energy at room per Hz, *NF* is the noise factor, and *B* is the bandwidth. Additionally, they define the signal-to-noise ratio as

(7) $$SNR=Pr+132-20log\frac{r}{\lambda }+CG+N,$$

where *Pr* is the reception power, *r* is the range, λ is the wavelength, and *CG* is the coding gain determined based on the SF. As in RPMA, the five used SFs depend on the following formula:

(8) $$SF=2^{k},k=\left[9:13\right];$$

so, the *CG* can be written as follows:

(9) GC=k×3.

Moreover, we can find the reception power by adding the noise level to the *SNR*.

(10) Pr=SNR+N.

As a result, we can express the reception power as

(11) $$Pr=Tx+132-20log\frac{r}{\lambda }+CG+N,$$

where *Tx* is the transmission power. In particular, the minimum reception power for RPMA equals −145, and the transmission power equals 21. Finally, we can estimate the coverage per SF, as illustrated in Table 2.

### 7.2. SFs Distribution Algorithms

**Figure 19:**
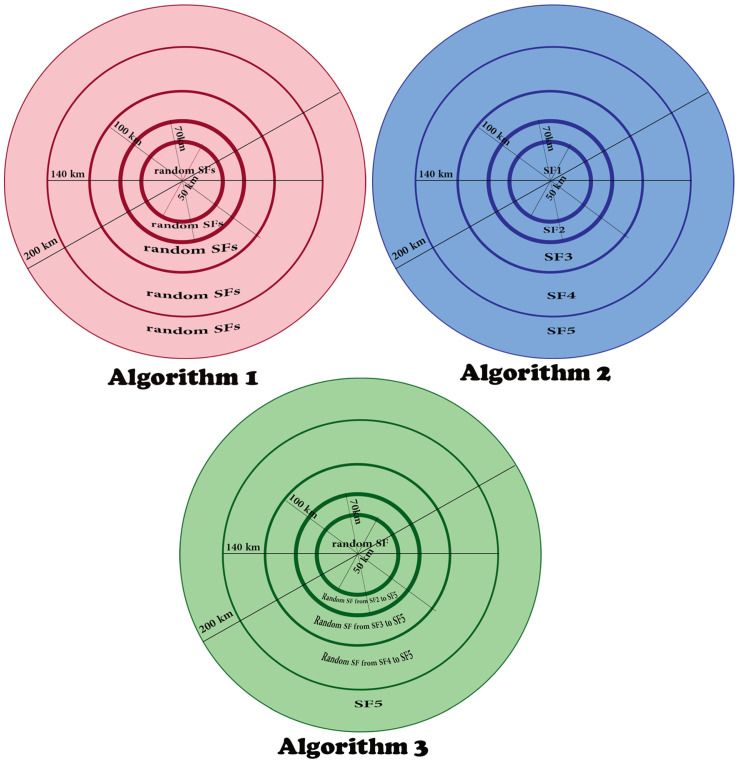
SFs distribution algorithms.

For an appropriate distribution of SFs, we divide the area around the access point into five circles with a radius of 25, 35, 50, 70, 100 km depending on the SFs coverage calculated in Table 1. Then we use three algorithms to distribute SFs in these rings. According to the first algorithm shown in Algorithms 1–3, the SFs are distributed entirely randomly. That means any node at any cell can choose any SF to transmit without considering the selected SF coverage. This scenario faces many packet losses, while in the second algorithm, a specific SF will be assigned to a particular cell based on the SF coverage. To clarify, SF1 will be assigned to the nearest cell to the access point, SF2 will be assigned to the second cell, SF3 will be assigned to the third cell, SF4 will be assigned to the fourth cell, and finally, SF5 will be assigned to the last and farthest cell from the access point. In other words, all nodes in the same cell will use the same SF to transmit. Finally, in the third algorithm, SFs’ distribution is partially random based on SFs coverage. In particular, the nodes in the first cell can select any SF to transmit since all SFs are eligible in the first cell. While the nodes in the second cell cannot select SF1 due to SF1 being unable to cover this area, thus nodes in cell 2 can select randomly from the set of eligible SFs ranging from SF2 to SF5. Similarly, in the remaining cells, where in the last cell, the nodes can only select SF5, which is the unique eligible SF as it has the largest coverage. These algorithms are described in Figure 19. In contrast, Algorithms 1–3 ensure packet delivery without losses due to SFs distribution based on their coverage.
**Algorithm 1:** SFs distribution Algorithm 1.**Input**: int Number of Nodes, Xa, Ya        /*Xa and Ya represent the Access point coordinates.*/**1** **for** *i = 1 to Number of Nodes* **do**(**2** Xi = rand [1 to 200] km**3** Yi = rand [1 to 200] km**4** $Distance_{i}=\sqrt{{\left(Xa-Xi\right)}^{2}+{\left(Ya-Yi\right)}^{2}}$**5** SF = rand [SF1 to SF5]

**Algorithm 2:** SFs distribution Algorithm 2.

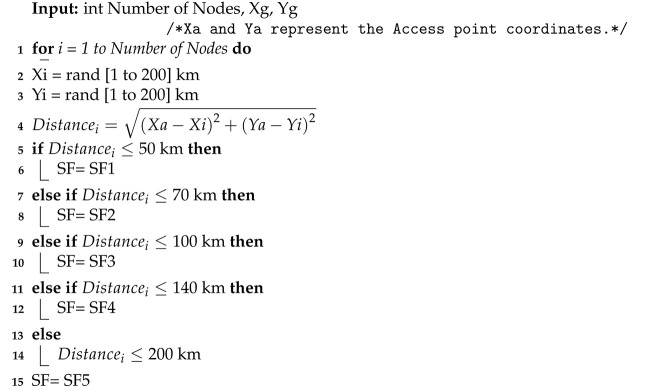



**Algorithm 3:** SFs distribution Algorithm 3.

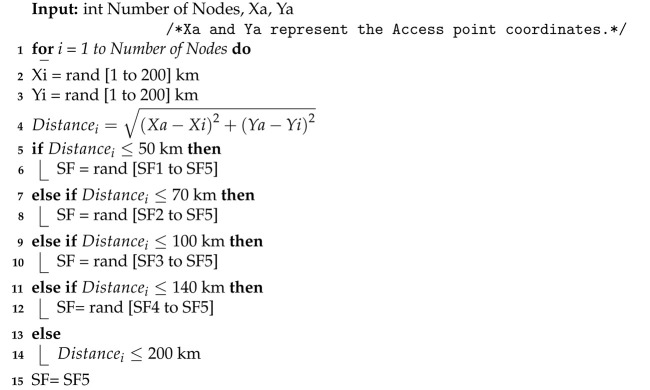



### 7.3. Simulation Results and Analysis

The simulation results of these three algorithms assess the impact of SFs distribution on RPMA scalability. Distributing SFs properly will help to further enhance the scalability of RPMA. We simulated these three scenarios in a network of 10,000 nodes transmitting on one day by adopting different numbers of channels [1, 10, 20, 30, and 38 channels] each time. Moreover, for accurate average results, each simulation scenario is repeated 100 times.

**Figure 20:**
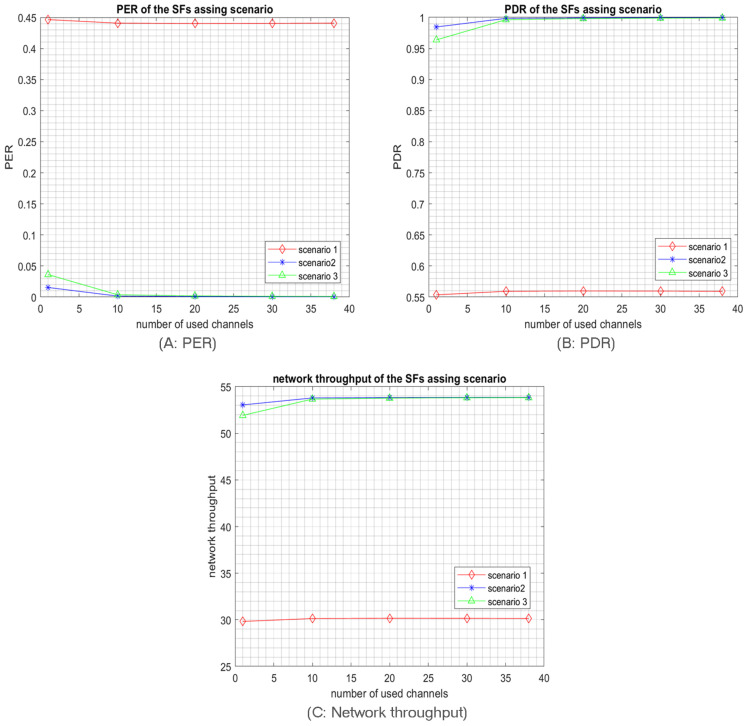
Simulation result of a network size of 10,000 nodes transmitting using low-traffic mode based on different SFs distribution algorithms.

In particular, the full randomness in the first algorithm leads to losing many packs before reaching the access points as the coverage of every SF is not respected. Thus, the PER is highly increased in addition to the collided packets. That is why the PER of scenario 1 is the highest, as shown in Figure 20. Hence, the PDR and the throughput for scenario 1 are the lowest.

**Figure 21:**
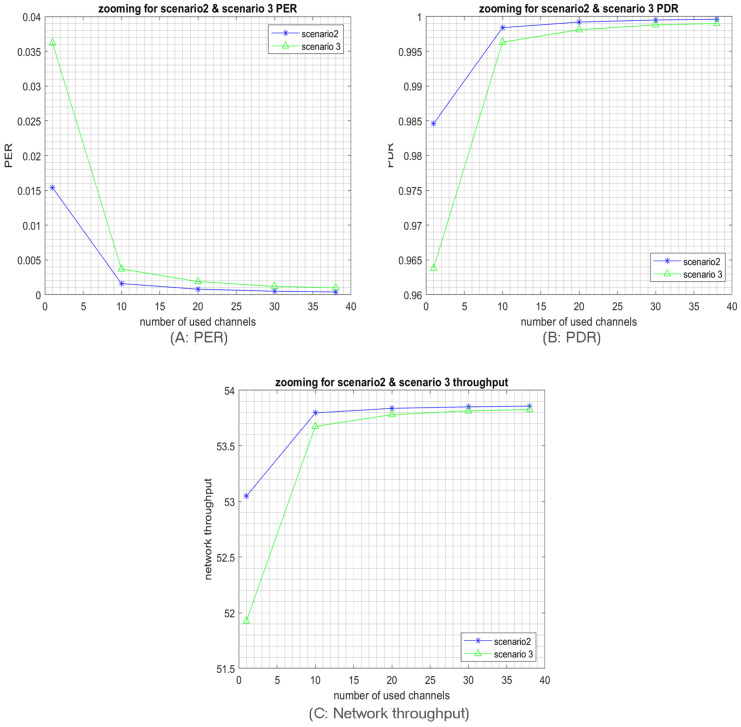
Zooming on simulation result for scenarios 2 and 3.

Although both Algorithms 2 and 3 consider the possible coverage for every SF, the results of Algorithm 2 are better than those of Algorithm 3, as shown in Figure 21. Accordingly, Algorithm 2 has the best SFs distribution. Indeed, in the second algorithm, the SFs are distributed in a way that respects the SFs coverage and ensures reduced collisions between packets. Recall that our previous simulation results (Section 5) and the mathematical analysis point to the high collision rates achieved by using high SFs. Please note that, according to Algorithm 3, SF4 and SF5 will be the most used ones as they will be selected in almost every cell. Whereas according to Algorithm 2, SF4 and SF5 are exclusively used in cell 4 and cell 5, respectively, as shown in Figure 19, which will increase the collision probability compared to the second algorithm, and hence the best results are achieved with Algorithm 2 as shown in Figure 21. Again, in all algorithms, we can notice the multichannel effect on RPMA scalability, where whatever the SFs distribution scenario, using multiple channels or exploiting all available channels in RPMA will enhance the network capacity 38 times. Table 3 provides a comparison between SFs’ distribution algorithms.

### 7.4. Comparison with LPWA Scalability Improvement Protocols

In this section, we will compare our proposed algorithms with the previous LPWA scalability improvement protocols that have been described in the related work section. Table 4 provides a summary of the achieved results and limitations of studies described in the related work section in addition to our three proposed algorithms. It is worth pointing out that our proposed algorithm 2 is the most efficient one as it highly reduces the PER, which makes it the most energy efficient protocol with the highest packet delivery ratio.

## 8. Conclusions

The higher demand for IoT applications has increased the number of connected devices. Thus, long-range-low-power network communication (LPWA) has emerged to satisfy IoT requirements. However, in terms of scalability, there is still room for improvement in order to cover the billions of connected devices that IoT applications need. One of the main issues is the duty cycle restrictions on the sub-GHz ISM band, in which most LPWA technologies work, along with the aloha based access mode. Contrarily, RPMA is an LPWA technology operating in the 2.4 GHz ISM band without duty cycle constraint, which helps RPMA to be a scalable LPWA technology. In this paper, we examined RPMA features and their impact on scalability.

First, we performed mathematical analysis to study RPMA scalability based on collision probability without considering the intentional delay feature in order to better study the impact of the relationship between the used SF and the number of available subslots. The results indicated that the collision probability will increase with the increase of the used SF. However, employing the multichannel feature helps to reduce collision probability, hence enhancing RPMA scalability, although the collision probability for higher SFs (SF4 and SF5) is still high.

Second, we conducted a simulation analysis by using MATLAB in two steps. Initially, we studied RPMA performance without considering adding the intentional delay, to investigate the effect of SFs on the collision possibility. The results showed that using higher SFs will increase the collision probability. As a main result, we found out that using only the three first small SFs will optimize the network performance. Moreover, utilizing all available channels will enhance RPMA scalability by up to 38 times. Then, we studied the effect of adding the intentional delay and RPMA’s ability to demodulate overlapping signals on the scalability. The results demonstrate that RPMA’s ability to demodulate overlapping signals helps to highly reduce the collision probability, even if a single channel connects the whole network, although using high SFs still affects the collision probability. However, employing all available channels will significantly enhance RPMA scalability by up to 38 times.

As a conclusion, taking advantage of the multichannel feature in RPMA will highly improve scalability and is considered a practical solution to satisfy the continuously increasing requirements of IoT applications. To reduce the effect of higher SFs on collision possibility, we proposed an SFs distribution algorithm that ensures efficient delivery with minimum collision. Dividing the area around the access point into five cells, and assigning each cell to the most appropriate SF that guarantees packet delivery, reduces the collision probability and hence a better network performance is achieved in terms of throughput and packet delivery ratio.

## Figures and Tables

**Table 1:** Comparison between key LPWA technologies.

Technology	Band	Range	Modulation	Data Rate	Topology
Dash7	Sub-GHz ISM band	2 km	Narrow-band modulation schema	167 kbps	Star or Tree
Telensa	Sub-GHz ISM band	2–3 km in urban 5–8 km in rural	UNB	62.5 bps (UL) 500 bps (DL)	Star
Weightless-W	TV whitespace	5 km in urban	DBPSK, BPSK, QPSK	1 kbps to 10 Mbps	Star
Weightless-N	Sub-GHz ISM band	5 km in urban	UNB, DBSK	100 pbs	Star
Weightless-G	Sub-GHz ISM band	2 km	QPSK, GMSK	200 bps to 100 kbps	Star
LoRa	Sub-GHz ISM band	15 km in urban 50 km in rural	CSS	50 kbps	Star or Mesh
Sigfox	Sub-GHz ISM band	10 km in urban 50 km in rural	DBPSK, GFSK	100 bps	Star
Ingenu-RPMA	2.4 GHz	16 km	RPMA-DSSS	78 kbps (UL) 19.5 kbps (DL)	Star

**Table 2:** The coverage per SFs.

SF	Coverage
SF1	50 km
SF2	70 km
SF3	100 km
SF4	140 km
SF5	200 km

**Table 3:** Comparison between SFs distribution algorithms.

SFs Destrbution Algorithm	SF Selection Machanisom	1 Channel Used			38 Channels Used		
		PER	PDR	Throughput	PER	PDR	Throughput
Algorithm 1	Fully random	0.4464	0.5536	29.8292	0.4407	0.5593	30.1379
Algorithm 2	Deterministic	0.0154	0.9846	53.0487	0.0004	0.9996	53.8575
Algorithm 3	Partly random	0.0362	0.9638	51.9256	0.0010	0.9990	53.8268

**Table 4:** Comparison with LPWA scalability improvement protocols.

Continued from Previous Page				
**Ref**	The Description	Technology	Achieved Results	Limitations
[6]	A framework depending on SDN to handle the inefficient radio resource allocation in LPWA technologies.	LPWA (LoRa-Sigfox)	Reduced number of collisions. Improve energy efficiency. Enhancing network throughput and scalability. Able to apply indifferent LPWA technologies.	Working better for small packet size. Do not give a practical solution for the Sigfox redundancy mechanism. Increased transmission delay.
[7]	Collision-prevention technique based on communication planning mechanism.	Long-range low-speed wireless IoT networks	Reduced number of collisions. Improve energy efficiency. Enhancing network throughput and scalability. Able to apply different LPWA technology.	Increase the complexity. The gateway is vulnerable to single-point failure Increased control overhead. Limited to the higher number of IoT devices. Limited to periodic translation.
[13]	Time and channel allocation mechanism.	Sigfox	Reduced number of collisions. Improve energy efficiency. Enhancing network throughput and scalability.	Do not provide information about end-to-end delay. The sub-GHz ISM duty cycle limitations restrict Sigfox scalability.
[8]	MAC layer protocol to enhance LoRaWAN scalability and reliability.	LoRaWAN	Reduced number of collisions. Enhancing network throughput and scalability.	Do not utilise SF’s orthogonality. the study conceded in a small size network (1000 nodes). The sub-GHz ISM duty cycle limitations restrict LoRa scalability.
[9]	SFs allocation schema.	LoRaWAN	Reduced number of collisions. Enhancing network throughput and scalability.	The study limited the use of 2 SFs while LoRa support 6 SFs. The study was limited to using only one channel. The sub-GHz ISM duty cycle limitations restrict LoRa scalability.
[17]	SFs distribution algorithm.	LoRaWAN	Reduced number of collisions. Enhancing network throughput and scalability.	Increased the transmission end-to-end delay. The sub-GHz ISM duty cycle limitations restrict LoRa scalability.
RPMA SFs distribution Algorithm 1	Randomness SFs assignment algorithm.	RPMA	Reduced number of collisions. Enhancing network throughput and scalability. No duty cycle limitations on RPMA.	Increased the number of lost packets, hence increasing the PER.
RPMA SFs distribution Algorithm 2	Deterministic SFs assignment algorithm.	RPMA	Highly reduced the PER. Vast enhancing network throughput and scalability. No duty cycle limitations on RPMA.	No limitations
RPMA SFs distribution Algorithm 3	Partly random SFs assignment algorithm.	RPMA	Reduced the PER. Enhancing network throughput and scalability. No duty cycle limitations on RPMA.	Do not respect the effect of SFs on transmission collisions

## Data Availability

The data presented in this study are available in the article.
